# Halogenated anthraquinones in breast cancer therapy: Structural modifications targeting VEGF-related angiogenesis pathways

**DOI:** 10.1016/j.jpha.2025.101538

**Published:** 2026-01-08

**Authors:** Chenyu Zhou, Murni Nazira Sarian, Xiaohui Tong, Rongchun Han, Theebaa Anasamy, Hamizah Shahirah Hamezah

**Affiliations:** a Institute of Systems Biology (INBIOSIS), Universiti Kebangsaan Malaysia, UKM, Bangi, Selangor, 43600, Malaysia; b School of Life Sciences, Anhui University of Chinese Medicine, Hefei, 230012, China; c School of Pharmacy, Anhui University of Chinese Medicine, Hefei, 230012, China; d Faculty of Medicine, Manipal University College Malaysia (MUCM), Jalan Padang Jambu, Bukit Baru, Melaka, 75150, Malaysia

**Keywords:** Breast cancer, Anti-angiogenesis, VEGF pathway, Structure-activity relationships, Halogenated anthraquinones

## Abstract

Halogenated anthraquinone derivatives have good potential in addressing the major clinical challenges associated with anthracyclines for breast cancer, including high toxicity, low selectivity, and drug resistance issues. By analyzing the structure-activity relationships of these compounds, the review highlights how specific structural modifications may enhance therapeutic efficacy. A comprehensive overview is also provided to categorize breast cancer progression across stages 0 to IV, along with therapeutic approaches for each of the three basic molecular subtypes, with placing particular focus on triple-negative breast cancer. This review details how anthraquinone derivatives modulate the vascular endothelial growth factor (VEGF)-related signaling pathways and regulate the expression of key proteins and chemokines, thereby inhibiting endothelial cell function during tumor angiogenesis, suppressing cancer cell migration and immune evasion in breast cancer. Studies on halogenated anthraquinone derivatives in anticancer applications between 2013 and 2025 are summarized, and the cutting-edge therapeutic strategies that have contributed to the development of anthraquinone derivatives are highlighted. The high approval rate of halogen-containing drugs released by the U.S. Food and Drug Administration in 2024, alongside their promising therapeutic efficacy in oncology, has once again drawn the attention of researchers. Overall, the contributions of halogen substitution to molecular stability, target specificity, and resistance to metabolic degradation highlight the significant potential of halogenated anthraquinone derivatives in the development of novel therapeutics for breast cancer.

## Highlights

• SAR analysis reveals that halogen substitutions improve molecular stability and selectivity of anthraquinone derivatives.
• Halogenated anthraquinones exhibit anti-angiogenic activity by modulating VEGF signaling axis, especially in TNBC.
• ROS induced by anthraquinone derivatives triggers cancer cell apoptosis or promotes host toxicity depending on the dosage.

## Graphical abstract

**Figure:**
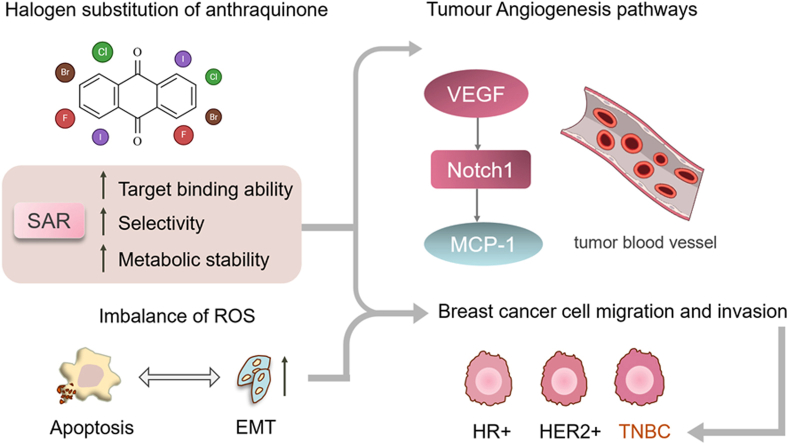

## 1 Introduction

Breast cancer is one of the most prevalent malignant tumors among women globally, and has been on the rise among young women in recent years. Especially in some low- and middle-income countries and regions, the survival rate of breast cancer patients is significantly lower than that of developed countries due to delayed diagnosis and lack of effective treatment [1]. Classic anthracycline drugs, such as doxorubicin (DOX) and epirubicin, remain central components of chemotherapy agents due to their significant antitumor activity. However, significant side effects such as cardiotoxicity, drug resistance, and non-specific cytotoxicity considerably limit their clinical efficacy and long-term safety [2]. While current studies on anthraquinone-based anticancer agents primarily focus on the inhibition of vascular endothelial growth factor (VEGF) and its downstream pathways such as nuclear factor kappa B (NF-κB) and phosphatidylinositide 3-kinases (PI3K)/protein kinase B (AKT), their regulatory effects on key components of the tumor angiogenesis remain largely underexplored [3]. With advancements in molecular subtyping and immunotherapy, research hotspots are gradually shifting from single anti-proliferative mechanisms to multi-dimensional therapeutic strategies targeting the tumor microenvironment, signaling pathways regulation, and drug structure modification.

## 2 Breast cancer

According to the latest data from the Global Cancer Observatory (https://gco.iarc.fr/), there were 18,094,716 new cases of cancer and 9,894,402 cancer-related deaths worldwide, and the top 5 most common cancers are breast cancer (12.5%), lung cancer (12.2%), colorectal cancer (10.7%), prostate cancer (7.8%), and stomach cancer (6%) [4]. As breast cancer is the most frequently occurring cancer, cancer statistics from the American Cancer Society (https://www.cancer.org/) in 2024 show that the breast cancer incidence rate has been gradually increasing by approximately 0.6% annually since the mid-2000s, which is more pronounced in women younger than 50 years, with a 1.1% annual increase compared to 0.5% for older women [5]. Breast cancer generally arises from reduced adhesion between breast epithelial cells and their uncontrolled proliferation, which often manifests as loss of epithelial integrity and tissue [6]. Through cell division, migration, differentiation, and other behaviors, malignant cells are produced that can break through the basement membrane as well as invade the tissue surrounding the breast and have the potential to metastasize to distant sites [7].

Based on the morphological and molecular characteristics, breast cancer can be roughly divided into three subtypes, including hormone receptor-positive (HR+), human epidermal growth factor receptor 2-positive (HER2+), and triple-negative breast cancer (TNBC). Approximately 70% of breast cancer cases are HR+ subtypes, that is, there are estrogen receptors (ER) or progesterone receptors (PR) on the surface of breast cancer cells [8]. HER2 is a protein that drives cell growth, and when overexpressed, it leads to rapid and uncontrolled proliferation of cells. This type of breast cancer tends to grow rapidly with a higher malignancy and is more prone to relapse or metastasis compared to other subtypes [9]. Among younger women, TNBC is more likely to occur, characterized by the simultaneous lack of expression of ER, PR and HER2 in breast tissues [10]. Differences in these subtypes determine the clinical manifestations and response to treatment of breast cancer.

### 2.1 Causes and metastasis of breast cancer

Diverse factors contribute to the development of breast cancer, mainly including family history, hormonal changes and environmental aspects. According to statistics, up to 10% of all breast cancers are hereditary. Mutations in the breast cancer susceptibility gene 1 (*BRCA1*) and *BRCA2* are strongly associated with the progression of breast cancer, and women carrying these mutations bear a significantly increased risk of developing breast cancer [11]. Studies have identified BRCA1 and BRCA2 as key tumor suppressor proteins involved in DNA repair. These genes facilitate the repair of damaged DNA, but in cases where repair is unsuccessful, cellular damage will occur. Highly penetrant mutations in these genes result in the loss of tumor suppressor function, thereby raising the risk of cancer [12].

In addition, estrogen levels in the body play a crucial role in the development of breast cancer, and estrogen is a major sex hormone in women, responsible for regulating the reproductive system, mammogenesis, and various other physiological functions [13]. In HR+ breast cancer, estrogen binds to ER such as ERα on breast cancer cells, initiating downstream cell signaling pathways and promoting cancer cell proliferation and survival [14]. Estrogen can also facilitate the production of pro-inflammatory factors, leading to changes in the tumor microenvironment and further driving the progression of cancer. Researchers found that hydroxylated estrogen accumulated in breast tissue triggered macrophage-dependent tumor metastasis by activating the nucleotide-binding oligomerization domain-like receptor family pyrin domain-containing 3 inflammasome and producing interleukin-1β [15]. The progression of breast cancer typically advances from early non-invasive carcinoma to locally invasive cancer. The hypoxic microenvironment within the tumor core prompts endothelial cells to initiate angiogenesis, facilitating access to blood and lymphatic vessels for metastatic dissemination. Clinically, tumor metastasis is commonly observed in bones, lungs, liver, and brain, which usually indicates a poor prognosis [16].

### 2.2 Clinical observations and disease progression

Breast cancer is graded into five stages based on factors such as the size of the tumor and the extent to which cancer has spread throughout the body, and the theoretical presentation is as stages 0, I, II, III, and IV, with higher numbers indicating more aggressive or spreading cancer [17] (Table 1). Carcinoma *in situ* is the earliest form of breast cancer, mainly including ductal carcinoma *in situ* and lobular carcinoma *in situ*. At stage 0, the cancer cells are confined to the breast ducts or lobules and have not yet invaded the surrounding tissues. The patient rarely detects the lump by self-examination, and it is often found incidentally during routine mammography [18]. As the disease progresses, patients may experience breast lumps, abnormal discharge from the nipple, orange peel-like skin changes of the breast, or other symptoms. The tumor gradually spreads to surrounding breast tissues, and multiple lymph nodes are involved. Therefore, axillary and supraclavicular lymph node enlargement is quite common [19,20]. For advanced breast cancer, also known as stage IV, distant metastatic symptoms such as bone pain and dyspnea may occur [21].

**Table 1:** Classification, pathological features, cell involvement, and treatment methods of breast cancer at stage 0, I, II, III, and IV [17]. Information regarding breast cancer cell subtypes was obtained from American Type Culture Collection (https://www.atcc.org).

Stage	Classification	Type of breast cancer cell involved	Pathological features	Treatment and drugs
0	*In situ*	MCF10DCIS.com	Non-invasive	Lumpectomy, mastectomy, radiation therapy, endocrine therapy (tamoxifen).
I	Early invasive	ER+: BT-474, BT-483, CAMA-1, HCC1428, HCC1500, MCF7, MDA-kb2, MDA-MB-175-VII, MDA-MB-361, MDA-MB-415, T47D, ZR-75-1, ZR-75-30; HER2+: AU-565, BT-474, HCC202, HCC1419, HCC1569, HCC1954, MDA-MB-175-VII, MDA-MB-361, MDA-MB-453, ZR-75-30; TNBC: BT-20, HCC38, HCC1395	Tumor size ≤2 cm, locally invasive, painless lump in the breast	Lumpectomy, mastectomy, radiation therapy, hormonal therapy (tamoxifen, letrozole, anastrozole, exemestane), chemotherapy (doxorubicin, epirubicin, pirarubicin, paclitaxel, docetaxel, nab-paclitaxel, cyclophosphamide), targeted HER2+ therapy (trastuzumab, pertuzumab), other adjuvant therapies followed by surgery.
II	Locally diffused		Tumor size: 2–5 cm, enlarged axillary lymph node	
III	Locally advanced		Tumor size ≥5 cm, extensive lymph node spread	
IV	Metastatic	HER2+: SK-BR-3, UACC-812 TNBC: BT-549, DU4475, HCC70 HCC1187, HCC1599, HCC1806, HCC1937, HCC2157, HCC2218, Hs 578T, MDA-MB-157, MDA-MB-231, MDA-MB-436, MDA-MB-468, UACC-893	Distant organs spread such as bones, liver, brain, and lungs	Chemotherapy (capecitabine, vinorelbine, eribulin), hormonal therapy (CDK4/6 inhibitors: palbociclib, ribociclib, abemaciclib), targeted BRCA-mutated therapy (PARP inhibitors: olaparib, talazoparib), immunotherapy (PD-1/PD-L1 inhibitors: atezolizumab, pembrolizumab), surgery, radiation; palliative care.

ER+: estrogen receptors; HER2+: human epidermal growth factor receptor 2-positive; TNBC: triple-negative breast cancer; CDK4/6: cyclin-dependent kinases 4 and 6; PARP: poly-adenosine diphosphate ribose polymerase; PD-1/PD-L1: programmed death-1/programmed death ligand-1.

Current treatment options contain surgery, radiation therapy, chemotherapy, endocrine therapy, and targeted therapy [22]. Due to the different stages of the disease, patients at early stage of breast cancer usually choose direct surgical resection. Meanwhile, locally advanced breast cancer patients should be treated with chemotherapy before surgery. Commonly used chemotherapy drugs include anthracyclines, taxanes and cyclophosphamide, and chemotherapy for stage IV is mainly used to control tumor growth, relieve symptoms, as well as improve the quality of life [23,24]. In addition, diverse subtypes need to be considered for treatment adjustment. HR+ breast cancer cells depend on hormonal signals for growth and proliferation, therefore, the treatment typically involves endocrine therapy, such as tamoxifen or aromatase inhibitors, to block hormone-receptor binding or reduce hormone levels in the body [25]. HER2-targeted therapy, such as trastuzumab, is rapidly advancing in recent years. However, some patients are not sensitive to or develop drug resistance, which is still an intractable challenge in treatment [26]. When resistance to initial treatment develops in the later stages of cancer, capecitabine and vinorelbine are used in combination with other drugs including trastuzumab to treat advanced HER2-positive breast cancer [27]. TNBC is considered the most aggressive subtype, which cannot be controlled by endocrine therapy or HER2-targeted therapy due to the lack of hormone receptors [28]. For the past few years, immunotherapy agents such as programmed cell death protein 1/programmed cell death ligand 1 (PD-1/PD-L1) inhibitors have been gradually applied to the treatment of TNBC, which can improve the undesirable responses caused by chemotherapy and the high risk of recurrence and metastasis [29]. For *BRCA1/2* mutant breast cancer, poly-adenosine diphosphate ribose polymerase (PARP) inhibitors remain an appropriate therapy [30]. As PD-1/PD-L1 immune checkpoint inhibitors combined with chemotherapy have shown good efficacy in clinical trials, research on breast cancer vaccines has also emerged and sparked controversy. Vaccine studies targeting α-lactalbumin, which is expressed in more than 70% of TNBC, have now entered Phase I clinical trials. However, the Phase III clinical trial of the E75 vaccine for patients with HER2-low expression, especially TNBC breast cancer, was terminated early because the interim analysis showed no significant improvement in disease progression-free survival [31]. So far, proven novel therapeutic strategies for TNBC remain limited, and extensive research is required to excavate more effective molecular targets.

## 3 Key signaling pathways in breast cancer

Angiogenesis is crucial for cancer progression, providing nutrients and oxygen to promote tumor growth and metastasis. This process is driven by factors such as hypoxia, VEGF, fibroblast growth factor, and transforming growth factor-α, which are critical in regulating endothelial cell behavior and immune responses within the tumor microenvironment [32].

### 3.1 Angiogenesis pathway

**Fig. 1:**
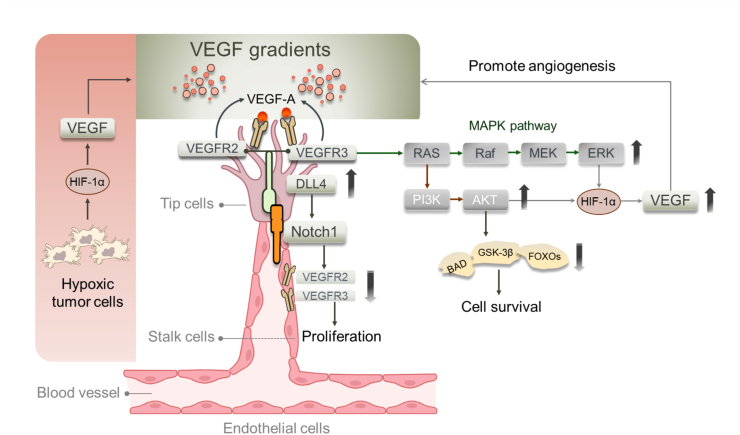
The schematic diagram illustrates the molecular mechanisms involved in vascular endothelial growth factor (VEGF) mediated angiogenesis, particularly within tumor microenvironments. HIF-1α: hypoxia-inducible factor 1-alpha; VEGFR: VEGF-receptor; Notch1: neurogenic locus notch homolog protein 1; DLL4: Delta-like ligand 4; RAS: rat sarcoma virus protein; Raf: Raf proto-oncogene serine/threonine-protein kinase; MEK: mitogen-activated extracellular signal-regulated kinase; ERK: extracellular signal-regulated kinase; MAPK: mitogen-activated protein kinase; PI3K: phosphatidylinositide 3-kinases; AKT: protein kinase B; BAD: B cell lymphoma-2 associated agonist of cell death protein, GSK-3β: glycogen synthase kinase-3 beta; FOXOs: forkhead box class O proteins. Created in BioRender. zhou, c. (2025) https://BioRender.com/jwic2kw.

Tumor angiogenesis refers to the process by which tumors promote the growth of new blood vessels from the existing vascular network by secreting various angiogenic factors like VEGF, platelet-derived growth factor, and fibroblast growth factor [33]. As the predominant angiogenic factor, VEGF binds to VEGF receptors (VEGFR) to activate downstream signaling pathways, including rat sarcoma (RAS)/mitogen-activated protein kinase (MAPK). RAS further activates the PI3K/AKT pathway by binding to its key effector protein, PI3K, and enhances PI3K activity through receptor tyrosine kinase mediation [34]. As the central node enzyme in downstream cell signaling pathways, AKT regulates the apoptosis of tumor vascular endothelial cells by inhibiting apoptosis-related factors such as B cell lymphoma-2 associated agonist of cell death protein, glycogen synthase kinase-3 beta (GSK-3β), and forkhead box class O, thereby promoting the proliferation, migration and tube formation of endothelial cells [35]. The VEGF family consists of seven members including VEGF-A, VEGF-B, VEGF-C, VEGF-D, placental growth factor, non-human genome encoded VEGF-E, and svVEGF. Among these, VEGF-A is the main mediator of tumor angiogenesis and plays an irreplaceable role in the angiogenesis of breast cancer [36]. Angiogenesis drives tumor expansion and invasion by providing adequate nutrients and oxygen to support rapid tumor growth, enabling tumors to spread from their original site to surrounding tissues and distant organs [37]. The Notch pathway also serves as a critical modulator of vascular branching in endothelial cells, orchestrating the balance of angiogenesis through its interaction with VEGF signaling. During angiogenesis, VEGF binds to VEGFR2 or VEGFR3 on endothelial cells, stimulating the growth of tip cells and activating delta-like ligand 4 (DLL4). Abundant VEGF is stored in the extracellular matrix, leading to an increased concentration of a gradient difference with the cell surface, thereby inducing the migration of tip cells. Other studies have also shown that this gradient is associated with differential VEGF secretion, which results from high expression of hypoxia-inducible factor 1-alpha (HIF-1α) in the hypoxic tumor center and extensive degradation of HIF-1α in the tumor periphery [38]. Similarly, the elevated levels of HIF-1α are also a result of PI3K/AKT pathway activation. As a ligand of the Notch receptor, DLL4 further activates the Notch signaling pathway in adjacent stalk cells. With the activation of neurogenic locus notch homolog protein 1 (Notch1), the expression of VEGFR2 or VEGFR3 on stalk cells is downregulated, allowing stalk cells to maintain proliferation without excessively differentiating into tip cells, which ensures the regulated growth of tumor vasculature [39] (Fig. 1). Additionally, epidermal growth factor (EGF) and its receptor, EGFR are instrumental in regulating the growth, survival, and metastasis of breast cancer cells. High expression of EGFR in breast cancer is associated with tumor aggressiveness, and targeted therapeutic strategies to inhibit EGFR have been shown to be effective in slowing tumor progression [40]. EGFR inhibitors such as gefitinib can reduce the proliferation and migration of breast cancer cells by blocking EGFR activation and downstream signaling, AKT/IκB kinase β/nuclear factor κB pathway, thereby inhibiting tumor progression. Besides, EGFR activation increases VEGF expression. Inhibition of EGFR not only directly inhibits the proliferation of tumor cells but also indirectly inhibits angiogenesis by reducing VEGF expression [41]. Hence, by modulating Notch and VEGF pathways, new targeted therapies may inhibit tumor angiogenesis and control tumor growth.

### 3.2 Tumor microenvironment and immune regulation

As a crucial chemotactic protein, monocyte chemoattractant protein-1 (MCP-1), also known as chemokine ligand 2, is involved in the progression of breast cancer microenvironment regulation. This process works primarily by recruiting immune cells, such as tumor-associated macrophages (TAMs), which normally show an M2-polarized macrophages (M2) phenotype in the breast cancer microenvironment and promote tumor growth and metastasis [42]. The chemokine MCP-1 attracts circulating monocytes and macrophages into tumor tissue and then they differentiate into TAMs. Cancer cells and the activated TAMs will secrete a large amount of MCP-1 and other cytokines, facilitating the polarization of macrophages into M2 subtype and strengthening this cycle [43].

The level of MCP-1 is usually regarded as a diagnostic marker for breast cancer metastasis. Significant overexpression of MCP-1 was observed in basal-like and claudin-low TNBC tissues, suggesting enhanced invasiveness without affecting cell proliferation which is associated with the p44/42 MAPK pathway activated by the chemokine receptor 2. To provide further proof, researchers inhibited MCP-1 expression in BT-549 cells using small interfering RNA (siRNA)/short hairpin RNA transfection and found that MCP-1 knockout weakened the aggressiveness of cancer cells and downregulated the expression of epithelial-mesenchymal transition (EMT) markers such as N-cadherin [44]. Similarly, MCP-1 induced phosphorylation of GSK-3β in MCF-7 cells and activated the expression of the EMT-related transcription factor Snail, significantly boosting migration and invasion. In addition, MCP-1 not only stimulated the secretion of VEGF but also indicated the early relapse of breast cancer by combining with VEGF status, which could further promote tumor angiogenesis [45]. Overall, the interactions between cancer-related factors highlight these proteins as promising candidate targets of anti-cancer and anti-metastasis in clinical applications.

## 4 Anthraquinone derivatives

**Fig. 2:**
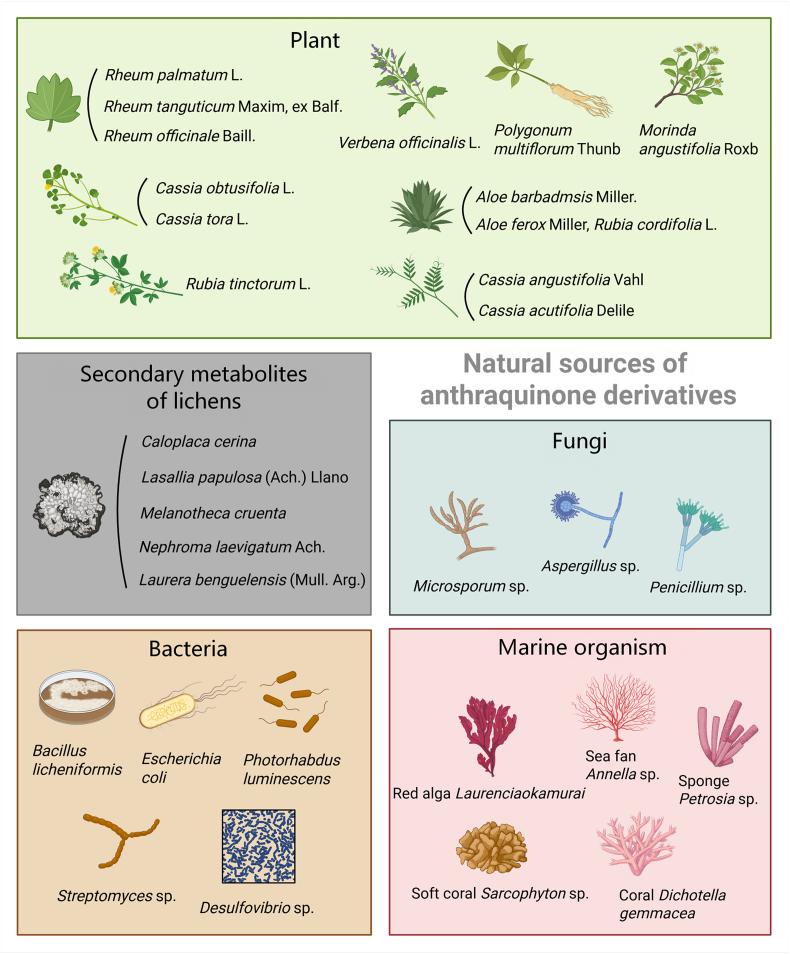
Anthraquinone derivatives from natural sources including plants, bacteria, fungi, marine organism, and secondary metabolites of lichens. Created in BioRender. zhou, c. (2025) https://BioRender.com/r12b219.

Anthraquinones are a class of organic compounds mainly obtained from abundant natural substances and chemical synthesis. Regarding natural sources, anthraquinones primarily exist in glycoside or free forms in diverse organisms [46], such as plants, bacteria, fungi, marine organisms and lichen metabolites [47] (Fig. 2). These organisms utilize intrinsic enzymatic reactions to synthesize anthraquinones through metabolic pathways such as the polyketide pathway including emodin and physcion [48], the shikimate pathway containing alizarin, and the mevalonate pathway involving various precursors [49]. The unique structure of anthraquinones is characterized by the anthracene ring and quinone group, comprising a fused three-ring system with two carbonyl (C=O) groups [50]. There are several commonly used methods for chemical synthesis of anthraquinone compounds, for example, synthesis of anthraquinone and its derivatives via the classical Friedel-Crafts acylation reaction, anthracene oxidation method, and multi-step chemical reactions including bromination, hydrolysis, and esterification [51,52].

Anthraquinone compounds are generally used as coloration in numerous fields like textiles, food, and cosmetics due to the highly conjugated aromatic system of anthraquinone molecules [53]. In addition, it has a wide range of pharmacological activities, including laxative effects, antioxidant, anti-inflammation, antimicrobial activity, and anti-cancer [49]. Among these compounds, the representative drug DOX has been adopted in clinical therapeutic for a long time, presenting distinct anti-breast cancer effects but alongside serious side effects containing multidrug resistance, cardiotoxicity, and weak targeting specificity eagerly need to be addressed [54].

### 4.1 Anticancer potential of halogenated anthraquinones

**Fig. 3:**
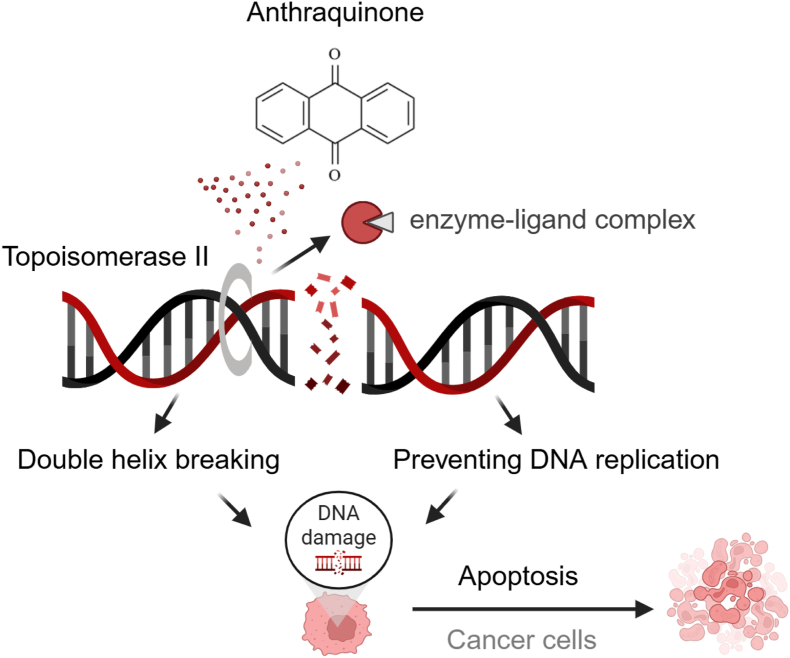
Mechanism of inducing cancer cell apoptosis by intercalating anthraquinone derivatives into DNA. Created in BioRender. zhou, c. (2025) https://BioRender.com/f7d0oiq.

Anthraquinone changes its physical and chemical properties through various chemical modifications including hydroxylation, methylation, sulfonation, glycosylation, carboxylation, amination, and halogenation, enhancing its applicability in dyes, electronic materials, and pharmaceuticals [55]. Anthraquinone and its derivatives are characterized by the aromatic ring planes, which are embedded in the DNA double helix through this structure and form a complex with isomerase II, further preventing DNA replication and transcription, thereby inducing apoptosis of cancer cells [56] (Fig. 3). Research on structure-activity relationships (SAR) has significantly advanced the progress of anthraquinones as anticancer agents. Among these, halogenated anthraquinone derivatives have garnered increasing attention due to their enhanced cytotoxicity, improved pharmacokinetic properties, and favorable interactions with key molecular targets. These halogen substituents can modulate the electronic structure, lipophilicity, and binding affinity of the parent compound, thereby influencing both activity and selectivity [46]. To better illustrate recent progress in this field, the halogenated anthraquinone compounds reported in the past decades (2013–2025) are summarized in Table 2 [[57], [58], [59], [60], [61], [62], [63], [64], [65]], along with their structural features and biological activities.

**Table 2:** Halogenated anthraquinones reported between 2013 and 2025 and their therapeutic potential as anticancer agents.

No.	Synonym	Structure	Label	Therapeutic potential	Refs.
1	1-(4-chlorophenylthio)anthraquinone		4-Cl-Ph-S	Cell viability of MDA- MB-231 and MCF-7 cells were decreased significantly by compound 1 treatment whereas no cytotoxic effect was observed in HUVECs, which is a healthy human endothelial cell line. Compound 1 was also more toxic to glioblastoma GBM02 cells than to peripheral blood mononuclear cells, with a SI value exceeding 1.33, and inhibited the migration of GBM02 cells at the 1/2 IC_50_ concentration of 30 μM.	[57,58]
2	2-chloro-N-(9,10-dioxo-9,10-dihydroanthracen-2-yl)acetamide		2-Cl-NHCOCH3	Western blot studies of protein levels in EU-1 ALL cell line showed that compound 2 upregulated the tumor suppressor p53 protein expression in a time-dependent manner.	[59]
3	3-(3-chloro-2-oxopropyl)-1,8-dimethoxyanthracene-9,10-dione		3-Cl-Propyl-1,8-OMe	Compound 3 exhibited cytotoxicity against EU-1 cells with an IC_50_ of 1.75 μM, and upregulated p53 expression in a time-dependent manner.	
4	1-chloroanthraquinone		1-Cl	The molecular docking analysis suggested that compound 4 acts as a good inhibitor of histone deacetylase 6 protein, which is associated with oral squamous cell carcinoma.	[60]
5	2-(chloro-N-(4,5-dihydroxy-9,10-dioxo-9,10- dihydroanthracen-2-yl)) acetamide		2-Cl-NHCOCH_3_-4,5-OH	IC_50_ values of compound 5 were determined to be 2.21 μM in HeLa cells, 0.83 μM in EU-1 leukemia cells, and 0.60 μM in MOLT-4 cells, which showed great potency comparable with DOX. Compound 5 also induced MDM2 protein degradation in severe combined immunodeficient mice by blocking the interaction between MDM2 and MDM4, thereby further activating p53 and inducing cell apoptosis in acute lymphoblastic leukemia.	[61,62]
6	2-[(chloromethyl)carbonylamino]-1,8-diethoxyanthracene-9,10-dione		2-CH_2_Cl-1,8-OEt	Compound 6 significantly inhibited viability in both MOLT-4 and EU-1 cell lines, with IC_50_ values being 0.077 and 0.28 μM.	
7	2-chloro-1,3,8-trihydroxy-6- (hydroxymethyl)-anthraquinone		2-Cl-1,3,6,8-OH-CH_2_OH	A reduction in cell viability was observed for compound 7 in both Kasumi-1 and Jurkat cells after 48 h treatment.	[63]
8	2-chloro-6-carboxy-1,3,8-trihydroxyanthracene-9,10-dione		2-Cl-6-COOH-1,3,8-OH	Compound 8 was identified in the bioactive extracts, which exhibited inhibitory activity against acute myeloid leukemia cells.	
9	3-bromo-1-hydroxy-9,10-anthraquinone		3-Br-1-OH	Compound 9 displayed cytotoxicity toward MCF-7 and MDA-MB-231 cell lines, along with good selectivity over the noncancerous MCF-10A cell line with a SI value of 2.78. It also induced cell cycle arrest in G1 phase of MCF-7 cells, and decreased the percentage of MDA-MB-231 cells migrating.	[64]
10	2-bromomethyl-1,3-dimethoxyanthraquinone		2-CH_2_Br-1,3-OMe	Compound 10 showed potent cytotoxicity against 5 cells lines: breast cancer (MCF-7), human uterine sarcoma (MES-SA), multidrug-resistant variant human uterine sarcoma (MES-SA/DX5), prostate cancer (DU145) and lung cancer (H460), with IC_50_ values in the range of 2–8 μM.	[65]
11	3-acetoxy-2-bromomethyl-1-methoxyanthraquinone		2-CH_2_Br-1-OMe-3-OAc	Compound 11 exhibited strong cytotoxicity against MCF-7 cells with IC_50_ of 6 μM and DU145 cells with IC_50_ of 5 μM.	
12	2-dibromomethyl-1,3-dimethoxyanthraquinone		2-CHBr_2_-1,3-OMe	Compound 12 showed anticancer activity with IC_50_ values were 10 and 7 μM for MES-SA and MES-SA/DX5 cell lines.	
13	2-bromomethyl-1-hydroxy-3-methoxyanthraquinone		2-CH_2_Br-1-OH-3-OMe	Compound 13 showed promising cytotoxicity with IC_50_ of 4 μM and enhanced the selectivity toward MES-SA cells.	
14	1-(trifluoromethylthio)anthracene-9,10-dione		1-SCF_3_	Treatment with compound 14 at IC_50_ concentration of 20 μM caused the loss of cytoplasmic content through the nuclear membrane breaking and the formation of cytoplasmic vacuoles in GBM02 cells. The migratory capacity of GBM02 cells was only 16 % when treated with compound 14 with 1/2 IC_50_ of 10 μM.	[58]
15	2-[(iodomethyl)carbonylamino]-1,8-bis(benzyloxy)anthracene-9,10-dione		2-CONHCH_2_I-1,8-OBn	Compound 15 showed not only the strongest cytotoxicity towards EU-1 (IC_50_ = 0.084 μM) and MOLT-4 (IC_50_ = 0.028 μM) cell lines, compared with DOX, but also the highest selectivity among tested cell lines, with 71-fold more selective toward Molt-4 and 24-fold toward EU-1 over p53-null leukemia K562 cell line.	[61]

SI: selectivity index; IC_50_: half maximal inhibitory concentration; ALL: acute lymphoblastic leukemia; p53: tumor protein p53; DOX: doxorubicin; G1: gap 1.

Chlorinated anthraquinones represent the largest group of derivatives reported in the past decade. 1-(4-chlorophenylthio)anthraquinone (**1**), bearing a 4-chlorophenylthio substitution, markedly reduced viability in MDA-MB-231 and MCF-7 breast cancer cells without harming normal HUVECs, and further suppressed migration of glioblastoma GBM02 cells. Similarly, 2-chloro-N-(9,10-dioxo-9,10-dihydroanthracen-2-yl)acetaide (**2**), a 2-chloroacetamide derivative, and 3-(3-chloro-2-oxopropyl)-1,8-dimethoxyanthracene-9,10-dione (**3**), with a chloro-oxopropyl side chain at the 3-position, both acted through upregulation of p53 in EU-1 leukemia cells. Simple chlorination at the 1-position, as in 1-chloroanthraquinone (**4**), was predicted by docking to inhibit HDAC6, a target relevant to oral squamous cell carcinoma. However, no supporting *in vitro* or *in vivo* evidence has yet been reported to validate this predicted activity. More complex chlorinated acetamide derivatives, such as 2-(chloro-N-(4,5-dihydroxy-9,10-dioxo-9,10- dihydroanthracen-2-yl)) acetamide (**5**) with additional 4,5-hydroxy groups, displayed strong potency in HeLa, EU-1, and MOLT-4 cells and promoted p53 activation via MDM2 degradation in acute lymphoblastic leukemia mice. Further optimization yielded 2-[(chloromethyl)carbonylamino]-1,8-diethoxyanthracene-9,10-dione (**6**), incorporating a chloromethyl-carbonylamino group and ethoxy substitutions, which showed sub-micromolar half maximal inhibitory concentration (IC_50_) values against leukemia cell lines. 2-chloro-1,3,8-trihydroxy-6- (hydroxymethyl)-anthraquinone (**7**), carrying hydroxyl groups at the 1-, 3-, and 8-positions of the anthraquinone scaffold, a hydroxymethyl substituent at the 6-position, and a chlorine atom at the 2-position, displayed growth inhibition against leukemia cells. 2-chloro-6-carboxy-1,3,8-trihydroxyanthracene-9,10-dione (**8**) shared the same substitution pattern at C-1, C-3, C-8 (hydroxyl groups) and C-2 (chlorine atom) as compound **7**, but differed at the C-6 position, where the hydroxymethyl group was replaced by a carboxyl group. Compounds **7** and **8** were both identified in bioactive extracts that exhibited inhibitory activity against leukemia cells; therefore, no quantitative IC_50_ values for the isolated single compounds are available. Within the brominated derivatives, structural modifications predominantly involved bromomethyl substitution. 3-bromo-1-hydroxy-9,10-anthraquinone (**9**), a 3-bromo-1-hydroxy analogue, exerted potent cytotoxicity against MCF-7 and MDA-MB-231 breast cancer cells while sparing normal MCF-10A cells. 2-bromomethyl-1,3-dimethoxyanthraquinone (**10**), with a 2-bromomethyl and 1,3-dimethoxy pattern, demonstrated broad activity across various cancer cell lines. More specific effects were observed for 3-acetoxy-2-bromomethyl-1-methoxyanthraquinone (**11**), incorporating a 2-bromomethyl and 3-acetoxy substitution, which strongly inhibited MCF-7 and DU145 cells, while 2-dibromomethyl-1,3-dimethoxyanthraquinone (**12**), featuring a dibromomethyl moiety, was active against both drug-sensitive and drug-resistant MES-SA cell lines. 2-bromomethyl-1-hydroxy-3-methoxyanthraquinone (**13**), combining a bromomethyl group with hydroxyl and methoxy substituents, enhanced selectivity toward MES-SA cells. Fluorinated anthraquinones were less common but structurally distinct. 1-(trifluoromethylthio)anthracene-9,10-dione (**14**), modified with a trifluoromethylthio group at the 1-position, displayed notable efficacy against GBM02 glioblastoma. Finally, among the collected anthraquinone derivatives, 2-[(iodomethyl)carbonylamino]-1,8-bis(benzyloxy)anthracene-9,10-dione (**15**), bearing an iodomethylcarbamoyl substituent and benzyloxy groups at the 1,8-positions, exhibited the most remarkable potency and selectivity in leukemia cell lines. Moreover, certain chlorinated anthraquinones, 7-chlorobisoranjidiol and 5-chlorosoranjidiol, have been identified as contributing to the remarkable phototoxicity. Although these two compounds have not been directly proven to possess anti-cancer activity, research suggested that they can induce and increase the generation of superoxide anion under light exposure, demonstrating excellent photosensitizing properties and having potential application value in the commonly used photodynamic therapy for destroying cancer cells [66].

### 4.2 Halogen-based structural modification of anthraquinone derivatives and SAR

**Fig. 4:**
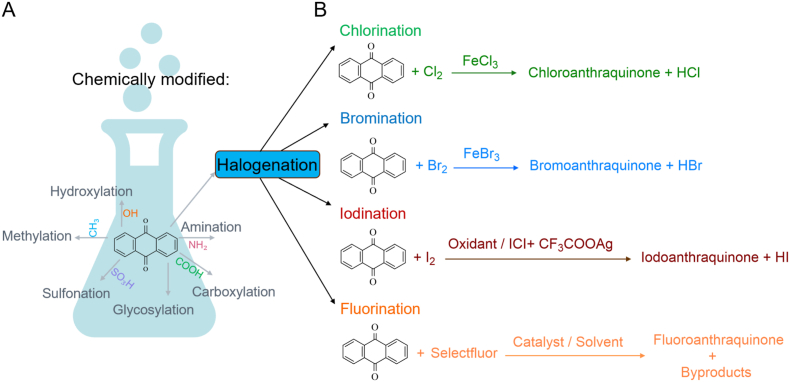
Schematic representation of common chemical modifications of anthraquinone and synthesis methods of halogenated anthraquinone derivatives. (A) Various chemical modifications of anthraquinone skeleton, including hydroxylation, amination, methylation, sulfonation, glycosylation, and carboxylation. (B) Four typical halogenation reactions using anthraquinone as substrate: chlorination, bromination, iodination, and fluorination. In the halogenation of anthraquinone derivatives, acetonitrile and dichloromethane are commonly used as solvents. In fluorination processes, polar aprotic solvents such as *N,N*-dimethylformamide and dimethyl sulfoxide are also employed. Created in BioRender. zhou, c. (2025) https://BioRender.com/akbfysf.

Halogenated anthraquinones can be obtained by chemical synthesis by substituting halogen atoms (Cl, Br, I, F) at different positions on the aromatic ring of the anthraquinone skeleton. This structural modification enables the generation of anthraquinone derivatives in chlorinated, brominated, iodinated, and fluorinated forms, expanding their chemical diversity and potential applications [67]. Notably, commonly used catalysts such as AgNO_3_ and Cu(OTf)_2_ can promote radical or single-electron transfer mechanisms in classical fluorination reactions. However, these catalysts have not been explicitly reported in fluorination studies of anthraquinone derivatives over the past two decades. In addition to conventional solvents like acetonitrile and dichloromethane, *N,N*-dimethylformamide is more suitable for electrophilic fluorination under mild conditions since the anthraquinone substrate has poor solubility. When higher temperatures (≥60 °C) reactions are required, dimethyl sulfoxide can be considered a solvent due to its thermal stability and ability to support polar environments [68]. Selectfluor is gradually consumed during the fluorination process, typically along with the production of byproducts such as 1,4-diazabicyclo[2.2.2]octane and hydrogen fluoride. Nevertheless, whether similar byproducts are generated during the fluorination of anthraquinones remains to be fully verified [69] (Fig. 4).

#### 4.2.1 SAR of chlorine and fluorine modification

According to the latest statistical analysis by the U.S. Food and Drug Administration (FDA) in 2024, among the 352 drugs approved by the U.S. FDA between 2018 and 2024, 108 are halogen-containing small molecule drugs. Moreover, chlorine and fluorine substitutions are more common in approved drugs [70]. From the perspective of SAR, the chlorine atom can form stronger hydrophobic interactions in aromatic or heterocyclic structures due to its relatively large atomic radius (99 p.m.), thereby enhancing the drug's target binding ability. In the anti-proliferative assay of chlorinated derivatives of 2′-hydroxychalcone against the MDA-MB-231 cell line, it was found that the presence of chlorine facilitated the compounds' passage through the lipid bilayer of the cell membrane, further enhancing their anti-proliferative activity, especially against invasive breast cancer cell lines [71]. Due to its availability and predictable reactivity, chloro-substitution exhibits broader applicability in SAR studies. According to the study that investigated the anticancer effects of introducing various halogenated acetyl groups at the R^2^ side chain of anthraquinone derivatives, compounds bearing α-chloroacetyl at the R^2^ position, especially compounds **5** and **6**, generally demonstrated strong cytotoxicity and higher selectivity toward wild-type p53 leukemia cells, suggesting a more defined target mechanism. In contrast, replacing this group with α-bromoacetyl or α-iodoacetyl did not lead to a significant enhancement in biological activity, despite their increased chemical reactivity, except for the iodoacetyl analogue compound **15**. Therefore, the suitability of both the substitution site and the chemical nature of the substituent is critical in the synthesis of new analogs. For instance, modifying the α-chloroacetyl group with bulkier or structurally different moieties, or excessively altering the side chain structure, may lead to decreased activity, as observed in compounds of 201, 215, 200, and 213 [61].

Fluorine, as the most electronegative element (Pauling = 3.98), can modulate the pKa value of drug molecules, affecting their solubility and binding affinity to target sites. As demonstrated in compound **14**, trifluoromethyl modification significantly enhanced the anticancer potential of anthraquinone derivatives against malignant tumor in the central nervous system, by increasing the electrophilicity and redox activity. Electrochemistry showed that compound **14** exhibited a positively shifted reduction peak in cyclic voltammetry, indicating that it undergoes reduction more readily. This was attributed to the strong electron-withdrawing effect of the trifluoromethyl group in the structure, which significantly enhanced the molecule's electron affinity and improved its reduction efficiency. Moreover, this group exerted an inductive effect that lowered the electron density at specific positions on the anthraquinone scaffold, and facilitated redox activity in both anodic and cathodic directions, which may promote oxidative stress within cells or induce apoptosis in cancer cells. The high electronegativity of the fluorine atoms conferred strong electrophilicity to the trifluoromethyl group, enabling stable binding to DNA bases. Moreover, the small size and lipophilicity of compound **14** may favor its interaction with specific DNA sites and its penetration into cancer cells [58]. In addition, fluorine substitution made the drug more difficult to be oxidized by the metabolic enzyme cytochrome P450 in the body, thereby extending the half-life and improving the metabolic stability of the drug [72].

#### 4.2.2 SAR of bromine and iodine modification

The bromomethyl group at position C-2 of the anthraquinone was found to play a substantial role in the cytotoxic activity of a class of brominated derivatives. Still, the effect varied with the amount of the bromine atom. Although compounds **10**, **11**, **12**, and **13** showed inhibitory potential against cancer cell lines, including breast cancer, uterine sarcoma, and prostate cancer, an additional bromine atom in the bromoalkyl group of dibromo derivative **12** seemed to reduce cytotoxicity compared to compound **10** in all the tested cancer cell lines, but increased the selectivity. As the calculated lipophilicity of the compounds showed, the highest predicted value for compound **12** (4.44 ± 0.64) could have been related to the increase in hydrophobic character, which was crucial in determining the capability of the compound to cross cell membranes [65]. This may be because cancer cell membranes typically contain a higher proportion of unsaturated fatty acids and cholesterol, which makes them more fluid and permeable. For instance, higher levels of phospholipids and fatty acids have been found in breast and colorectal cancer cells. This increased fluidity allows hydrophobic molecules to interact more readily with and penetrate the cancer cell membrane, thereby leading to targeted cytotoxicity [73]. However, disadvantages such as lower solubility and reduced absorption efficiency due to high hydrophobicity remain key challenges that need to be balanced in future drug development.

Although iodination is rarely reported in the context of enhancing the anticancer activity of anthraquinone derivatives, it has shown great promise in advancing breast cancer diagnostics. A 2024 study demonstrated that rhein-coated gold nanoparticles labeled with ^124^I possessed strong potential as targeted positron emission tomography imaging agents. These nanoparticles exhibited notable accumulation in ERα-positive MCF-7 breast cancer cells, indicating efficient targeting capabilities. Furthermore, imaging studies in a mouse model revealed high tumor uptake and clear visualization, supporting the feasibility of using ^124^I-labeled rhein-gold nanocomposites as a powerful tool for early detection and imaging of breast cancer [74].

Different substitution sites of halogen atoms also showed uneven selectivity for cytotoxicity. Compounds **10**–**13**, modified with brominated groups at position C-2 of the anthraquinone, showed enhanced cytotoxicity against various cancer cells. In addition, the thermodynamic stability of halogenated anthraquinones, including chlorine, bromine and fluorine derivatives, was analyzed using density functional theory. The results showed that halogen substitution at lateral positions such as 2, 3, 7, and 8 significantly enhanced molecular stability, while substitution at peri positions like 1, 4, 6, and 9, or simultaneous substitution at adjacent positions might have led to electronic repulsion. This structural trend is favorable for improving the metabolic stability and binding consistency of drugs, providing a theoretical basis for the design of anthraquinone-based pharmaceuticals [75]. Therefore, the great potential of halogen atoms to enhance drug potency and selectivity gives them broad prospects in the development and application of novel anticancer drugs. Researchers have attempted to promote such modifications to improve their binding affinity to targets such as VEGF and inhibit various enzymes including protein kinases, topoisomerases, and matrix metalloproteinases, therefore enhancing biological activities [56].

#### 4.2.3 Comparison of physicochemical and biological properties across halogen substitutions

Halogen substitution exerts profound effects on the physicochemical characteristics and biological activities of anthraquinone derivatives (Table 3). Reported data and examples indicate heavier halogens increase lipophilicity and organic phase solubility. A study on halogen substituted anthraquinones used as organic cathode materials reported that substitution with chlorine, bromine, or iodine decreased the solubility of anthraquinones in battery electrolyte, whereas fluorine substitution significantly increased their solubility [76]. This is because heavier halogens provide a larger and more polarizable surface area, thereby enhancing the hydrophobicity of the compound. In particular, iodine, due to its high polarizability, can form stronger halogen bonds or halogen-oxygen contacts, which allow the molecules to pack more tightly in the crystal lattice. As a result, the lattice energy increases, making it more difficult for water molecules to disrupt the crystal and drive the compound into solution, thus leading to reduced solubility [77]. Fluorine, being the smallest halogen in terms of atomic radius, exhibits effects that vary depending on whether it is introduced as a small atom on an aromatic ring or as part of a perfluoroalkyl group. Small-ring F atoms can increase the dipole moment or alter local electrostatics, thereby facilitating interactions of certain anthraquinones with water and improving aqueous solvation [78]. In contrast, multiple fluorine atoms or perfluoroalkyl chains, such as the SCF_3_ group in compound **15**, enhance lipophilicity, as they generate low-polarity fluorine surfaces that dissolve well in non-polar media [79].

**Table 3:** Effects of different halogen substitutions on the physicochemical properties and biological activities of anthraquinone derivatives collected in this review.

Halogen	Water solubility	Lipophilicity and organic solubility	Cytotoxicity	Selectivity
Cl	Typically decreases aqueous solubility relative to F.	Increases organic solubility and hydrophobicity.	Enables alkylating or chloroethyl anthraquinones that are highly cytotoxic via DNA reaction.	Shows relatively high tumor selectivity when targeting rapidly dividing cells.
Br	Generally further decreases water solubility compared with Cl.	Exhibits greater lipophilicity and stronger solid-state interactions than Cl.	Often increases cytotoxicity in quinones and related scaffolds compared to Cl.	Provides a favorable balance between cytotoxicity and selectivity.
I	Causes a pronounced decrease in aqueous solubility.	Tends to show higher organic solubility among halogens and stronger solid stabilization.	Demonstrates relatively strong potency in quinone cores.	Displays strong cytotoxicity but is often accompanied by notable host cell toxicity.
F	Often neutral to slightly increased compared with heavier halogens.	Perfluoroalkyl groups markedly increase lipophilicity.	Limited data in anthraquinone series evidence.	Insufficient anthraquinone-specific data.

Anthraquinone-related quinone systems exhibit a halogen-dependent potency trend, where larger and more polarizable halogens typically confer higher cytotoxicity, though specific data on anthraquinone compounds remain limited. Among halogenated benzoquinones, iodine-substituted analogs are identified as the most toxic, while brominated compounds display greater cytotoxicity than their chlorinated counterparts, which has demonstrated a clear cytotoxicity ranking in studies on halogenated benzoquinones (HBQs) [80]. After 24 h exposure on human urothelial SV-HUC-1 cells, the IC_50_ values were 137 ± 2 μM for 2,6-dichloro-1,4-benzoquinone, 139 ± 2 μM for 2,6-dibromo-1,4-benzoquinone, and 85 ± 1 μM for 2,6-diiodo-1,4-benzoquinone, indicating that iodine substitution conferred the strongest toxicity [81]. Similar observations were reported in Chinese hamster ovary cells, which were treated with the halogen substitutions of HBQs, demonstrating that diiodo-HBQs > dibromo-HBQs > dichloro-HBQs [82]. Moreover, cytotoxicity assays of compound **15** indicated that iodinated anthraquinones exhibited potent cytotoxicity with relatively high selectivity in specific cancer cell lines, although data in other cancer models remain limited. In parallel, it has also been reported that iodinated emodin displayed enhanced antiviral potency against human coronavirus HCoV-NL63, but this was accompanied by increased toxicity toward the host kidney derived Vero cell line [83]. While this does not provide direct evidence of poor cellular selectivity, the potential adverse effects associated with cytotoxicity remain a critical challenge for the therapeutic application of such compounds across various diseases. The cytotoxicity of chlorine-substituted anthraquinones is highly mechanism dependent. Simple chlorine substitution on the anthraquinone scaffold typically confers only moderate potency, primarily by increasing lipophilicity and membrane permeability. In contrast, when chlorine atoms are incorporated into alkylating moieties, such as chloroethylamino or chloromethyl groups, these derivatives can achieve nanomolar cytotoxicity through covalent DNA alkylation and cross-linking. Several 1,4-disubstituted chloroethylaminoanthraquinones were reported to exhibit potent cytotoxicity against human ovarian cancer A2780 cells, with IC_50_ values ≤ 40 nM, and such chloroalkyl anthraquinones were considered to display high tumor selectivity in rapidly proliferating cells while carrying an inherent risk of off-target DNA damage in normal tissues, although in recent years few studies have provided direct evidence to support this viewpoint [84]. This mechanistic distinction is also reflected in their target-dependent selectivity. The substitution of the chlorine can affect binding affinity to specific molecular targets in cancer cells, such as PARP-1, providing insights for designing more selective inhibitors [85].

#### 4.2.4 Translational insights from U.S. FDA-approved halogen-containing drugs for optimizing halogenated anthraquinones

Although none of the U.S. FDA-approved halogen-containing anticancer small molecules share a highly similar anthraquinone scaffold, their halogen utilization strategies provide valuable guidance for the translational optimization of halogenated anthraquinones. For instance, vorasidenib is an oral agent approved for the treatment of WHO grade II astrocytoma or oligodendroglioma harboring susceptible isocitrate dehydrogenase-1 (*IDH1*) or *IDH2* mutations. SAR studies revealed that replacing the CF_3_ group at the 6-position with polar groups reduced potency dramatically (IC_50_ = 201–1749 nM), whereas replacement with Cl partially restored activity (IC_50_ = 32 nM). The ethylcyclopropyl group was then replaced with a trifluoroethylcyclopropyl group, ultimately leading to the synthesis of vorasidenib. The combined introduction of Cl and F substituents reduced the IC_50_ against glioma cells to the low nanomolar range (IC_50_ = 0.25–7 nM). The co-crystal structure with the IDH1-R132H dimer reveals that the chlorine atom forms a halogen bond with the carbonyl group of D273, while the CF_3_ substituents interact with the carbonyl oxygens of Q277 and V255 residues. The halogens in the structure are precisely arranged on aromatic or heteroaromatic rings, utilizing halogen bonds and hydrophobic interactions to enhance binding selectivity. These key interactions contribute to the enhanced potency of vorasidenib [70,86]. In contrast, most halogenated anthraquinones collected in this review are modified with DNA-reactive alkyl substituents, typically alkyl halides or α-haloamides on their side chains, including compounds **2** and **5** (CO-CH_2_Cl), **10** and **11** (CH_2_Br), **12** (CHBr_2_), and **13** (CH_2_Br). These substituents are usually positioned close to nucleic acids after the anthraquinone scaffold engages DNA through noncovalent interactions such as intercalation or groove binding, making them highly prone to alkylation of nucleobase nucleophilic sites or even further cross-linking [87]. While this direct mode of DNA attack is highly effective, it is also accompanied by substantial host toxicity.

Similarly, another U.S. FDA-approved drug inavolisib, a therapy for PI3K p110α-mutated advanced or metastatic breast cancer, was synthesized by optimizing the structure of the reported PI3Kα inhibitor, GDC-0326. The parent scaffold had excellent affinity (PI3Kα *K*_i_ = 0.026 nM) but poor permeability (*P*_app_ = 0.3 × 10^−6^ cm/s). To optimize pharmacokinetic properties, a CHF_2_ group was installed onto the oxazolidinone moiety, which improved both activity (PI3Kα *K*_i_ = 0.053 nM) and permeability (*P*_app_ = 4.3 × 10^−6^ cm/s). Subsequent truncation of the pyrrolidine ring and replacement of CHF_2_ with CF_3_ demonstrated substantial improvement in selectivity (171-fold over PI3Kδ) but resulted in slightly reduced biochemical activity (PI3Kα *K*_i_ = 0.095 nM). The final switch back to CHF_2_ yielded inavolisib, as this group was able to form non-classical hydrogen bonding with Ser774 of PI3Kα, thereby enhancing potency (PI3Kα *K*_i_ = 0.034 nM), improving 361-fold selectivity over PI3Kδ, and stabilizing target engagement [70,88]. Compound **14** also introduced a strongly electronegative SCF_3_ group onto the anthraquinone scaffold, which decreased the electron density of the core and thereby facilitated the reduction process, leading to excessive reactive oxygen species (ROS) generation and subsequent cellular damage. Although both strategies exploit the electronegativity and polarization effects of fluorine substituents to modulate molecular behavior, the action of inavolisib relies on precise protein–ligand recognition, in which fluorine interacts directly with amino acid residues. Specifically, inavolisib selectively binds to the ATP-binding site of PI3Kα, promotes degradation of the catalytic subunit p110α, thereby suppressing downstream AKT phosphorylation and inducing apoptosis in cancer cells such as MCF-7. In most studies on the anticancer activity of halogenated anthraquinones, inhibition of the AKT pathway is not uncommon. However, mutations or amplifications of PI3K are the main causes of drug resistance, and the multiple PI3K isoforms are the major source of side effects. By resolving the co-crystal structure of PI3Kα with lead compounds, researchers identified PI3Kα-specific residues Q859 and H855, and introduced side-chain substituents capable of forming multiple hydrogen bonds with Q859, thereby significantly enhancing PI3Kα selectivity. Furthermore, the incorporation of an acyclic alanine carboxamide improved selectivity against other PI3K isoforms such as PI3Kδ. Unlike traditional inhibitors that merely block activity, the structure of inavolisib is designed to specifically bind to the mutant p110α/p85β protein complex. This binding triggers the cell's ubiquitination machinery, which tags the mutant p110α for degradation by the proteasome [89]. By physically removing the mutant protein, inavolisib prevents pathway reactivation and resistance arising from secondary mutations at the inhibitor binding pocket, which is a common issue with other candidate drugs. For example, anthraquinone derivatives rely on redox cycling to generate ROS, which can be counteracted by cellular antioxidant responses such as nuclear factor erythroid 2-related factor 2 activation and increased glutathione levels [90]. Inspired by the strategy of inavolisib, structural optimization of halogenated anthraquinones to not only generate ROS but also promote degradation or signaling blockade through specific protein interactions may help reduce resistance. On the other hand, the iterative fine-tuning of CHF_2_ and CF_3_ groups on the aromatic ring was not merely intended to increase cytotoxic potency, but rather to improve selectivity and membrane permeability, thereby helping the drug achieve an optimal balance among activity, selectivity, and metabolic stability. This is exactly the focus currently lacking in most halogenated anthraquinone SAR research.

## 5 Regulation of molecular targets for breast cancer therapy by anthraquinone derivatives: Inspiration from emerging therapies

### 5.1 Anthraquinone derivatives targeting VEGF signaling in breast cancer angiogenesis and metastasis

Breast tumor development largely depends on the angiogenic sprouting and expansion of the vascular network, and VEGF-A and its receptor VEGFR are key molecular players in the angiogenic passageway [91]. Numerous laboratory findings indicated that anthraquinone derivatives demonstrated antitumor angiogenesis potential. The classical compound emodin (1,3,8-trihydroxy-6-methylanthracene-9,10-dione) repressed the proliferation of the MCF-7 cell line in a dose-time- dependent manner and significantly decreased the expression levels of VEGF-A and VEGFR-2 [92]. Similarly, both aloe-emodin (1,8-dihydroxy-3-(hydroxymethyl) anthracene-9,10-dione) and emodic-acid (4,5,7-trihydroxy-9,10-dioxoanthracene-2-carboxylic acid) reduced the expression of VEGF protein and matrix metalloproteinase-9 messenger RNA, the latter of which is regulated downstream of the NF-κB pathway, thereby weakening the invasion and migration capabilities of 4T1 cells [93]. Under hypoxic conditions, rhein (4,5-dihydroxy-9,10-dioxoanthracene-2-carboxylic acid) down-regulated VEGF_165_ and EGF levels in MCF-7 and MDA-MB-435s cells, then inhibited VEGF-induced angiogenesis by suppressing the activation of the PI3K/AKT/extracellular signal-regulated kinase (ERK) pathway [94]. Likewise, studies on zebrafish embryos have shown that 1,8-dihydroxy-3-methoxyanthraquinone disrupted hypoxia-induced HIF-1α stability without causing off-target cytotoxicity and subsequently inhibited VEGF-mediated tumor angiogenesis. Moreover, the downregulation of PI3K/AKT and Raf proto-oncogene serine/threonine-protein kinase/ERK pathways by this derivative also suppressed tumor growth [95].

The cytoskeleton, composed of microtubules, intermediate filaments, and actin filaments (F-actin), is essential for maintaining cell shape, migration, and invasion. Researchers employed the Notch1 siRNA plasmid transfection technique to confirm that Notch1 silencing not only affected the proliferation of MDA-MB-231 cells but also impaired their migration, due to the reduction of F-actin polymerization [96]. In addition, Notch1 enhanced resistance to anthracyclines such as DOX in breast cancer by activating the AKT pathway and promoting EMT, hence upregulating drug-resistant proteins such as multidrug resistance-associated protein 1, which suggests that suppressing Notch1 signaling may increase sensitivity to chemotherapy drugs [97]. Unfortunately, no literature currently supports that halogenated anthraquinones inhibit breast cancer cell migration via the Notch1 pathway, nor is there experimental evidence proving that anthraquinone derivatives affect the chemokine MCP-1, a key marker associated with TNBC. This research gap is undoubtedly an obstacle to solving the problem of metastasis in breast cancer.

### 5.2 Promising conjugated therapies and mechanisms in breast cancer treatment

Currently, traditional chemotherapy combined with immunotherapy remains the preferred clinical approach for breast cancer treatment. Typically, the tumor lesions shrink after several treatment cycles, but resistance soon develops and results in recurrence or metastasis due to ineffective long-term control [98]. Moreover, chemotherapy drugs often cause damage to normal cells, leading to multiple side effects and considerable suffering for patients. Monoclonal antibodies targeting tumor cell surface antigens appear to offer a potential breakthrough to address these challenges. To overcome the limitations of monoclonal antibodies' weak cytotoxicity and the lack of specificity in traditional cytotoxic drugs, antibody-drug conjugates (ADCs) have emerged as cutting-edge therapeutic compounds in oncology [99]. These ADCs have attracted widespread research interest and technological advancement. By linking chemotherapeutic agents to antibodies, the drugs can be delivered directly to tumor cells, ensuring targeted and precise drug release within the tumor [100]. This technology was first applied in HER2+ breast cancer, with some drugs already approved for clinical use, such as trastuzumab deruxtecan (T-DXd) and trastuzumab emtansine (T-DM1). In particular, the T-DXd shows greater potential, with the results of the DESTINY-Breast03 trial suggesting that it significantly prolonged median progression-free survival and overall survival in patients with HER2+ metastatic breast cancer, and reduced the risk of death by 36 % [101]. Furthermore, sacituzumab govitecan, marketed as Trodelvy, has also shown promising results in clinical treatment specifically targeting advanced TNBC. The tumor-associated transmembrane signal transducer Trop-2, which is overexpressed in various TNBC cell lines, is a specific target of Trodelvy. It is linked to the topoisomerase I inhibitor and destroys cancer cells by interfering with DNA replication [102].

In addition, when *BRCA1/2* gene mutations are present in cells, the deficiency in homologous recombination repair renders the cells incapable of effectively repairing DNA double-strand breaks. The application of PARP inhibitors blocks the repair of single-strand breaks, leading to the accumulation of double-strand breaks during DNA replication. The combination of these defects results in a synthetic lethality effect, which intensifies tumor cell death [103]. Currently, third-generation PARP inhibitors are widely used in the clinical treatment of ovarian cancer and TNBC. Olaparib, the first PARP inhibitor approved by the U.S. FDA for the treatment of advanced breast cancer, has been shown to significantly reduce the risk of disease progression or death in patients with *BRCA1/2*-mutated advanced breast cancer, with even greater efficacy observed when used in combination therapy. The combination of olaparib, durvalumab, and paclitaxel demonstrated a superior pathological complete response rate compared to standard neoadjuvant chemotherapy in patients with HER2− breast cancer, particularly in the high-risk HR−/HER2− subtype characterized by high MammaPrint scores (64% vs. 22%) [104]. The newly approved investigational anticancer drug HS-10502 is a PARP1 inhibitor. HS-20089 and HS-20093 for injection are ADCs targeting B7-H4 and B7-H3, respectively. Among them, B7-H4 is commonly overexpressed in PD-L1-negative tumors, such as TNBC, and plays a role in helping cancer cells evade immune surveillance [105]. These advancements highlight the advantages of conjugated therapies, which combine targeted delivery with potent cytotoxic effects to improve therapeutic efficacy while minimizing off-target toxicity.

Some halogenated anthraquinones in their free-drug form, particularly alkyl-substituted derivatives, exhibit extremely potent anticancer activity. For instance, 1,4-chloroethylamino-anthraquinones showed IC_50_ values below 40 nM in A2780 cells. However, such potency is commonly associated with DNA alkylation, which induces helix distortion and interstrand cross-linking, thereby conferring strong cytotoxicity but also raising concerns of host toxicity and a narrow therapeutic window [84]. Classical anthraquinone-based drugs, such as DOX, daunorubicin and mitoxantrone, despite their powerful efficacy, possess a systemic and broad toxicity profile. Non-selective cytotoxicity results in side effects typical of chemotherapy including nausea, myelosuppression and cardiotoxicity [106]. ADCs are essentially the most selective among the aforementioned treatment strategies, particularly when non-cleavable linkers are used to connect the antibody and payload. This stable linker releases the payload only after the antibody is internalized and degraded by the tumor cell, thereby reducing the amount of free toxin and minimizing the risk of systemic side effects [107]. According to a meta-analysis of clinical trials involving 2016 breast cancer patients treated with T-DM1, compared to respective control groups receiving trastuzumab, anthracyclines, taxanes, and other conventional chemotherapy strategies, patients in T-DM1-treated groups experienced lower rates of diarrhea (17% vs. 62%), as well as reduced incidence of nausea (40% vs. 45%) and vomiting (20% vs. 29%) [108]. However, since the payload is the core component responsible for killing cancer cells, it inevitably carries potential side effects. ADCs often utilize highly potent payloads, such as monomethyl auristatin E, which can lead to peripheral neurotoxicity, manifesting as numbness, tingling, and similar symptoms in patients [109]. Nevertheless, compared to traditional chemotherapy, these adverse effects are more predictable and primarily determined by the specific type of payload used, making them generally more manageable. On the other hand, ADCs face challenges including complex and multi-factorial resistance development. One common mechanism is antigen loss, where tumor cells downregulate or completely stop expressing the target antigen, rendering the payload unable to bind and initiate its cytotoxic effect. Even when antigen expression is retained, defects in internalization or intracellular trafficking can hinder the ADCs from reaching lysosomes, preventing payload release. Furthermore, upregulation of drug efflux pumps, such as P-glycoprotein, can expel the payload from cancer cells before it exerts the effect [110]. In contrast, certain anthraquinone derivatives have been shown to inhibit P-glycoprotein, particularly in the context of overcoming multidrug resistance in cancer cells. For instance, emodin decreased P-glycoprotein expression via the MAPK/activator protein-1 pathway through cyclooxygenase-2 inhibition in Caco-2 cell lines [111]. In a study reporting the antiviral activity of halogenated emodin derivatives against severe acute respiratory syndrome coronavirus 2, it was noted that all compounds bearing two halogen atoms (I, Br, or Cl) simultaneously at positions 2 and 4 exhibited activity against methicillin-resistant *Staphylococcus aureus*. Moreover, 4-chloroemodin was found to significantly inhibit common drug-resistant strains such as vancomycin-resistant Enterococcus through a dual antibacterial mechanism that targets both the bacterial cell membrane and DNA [83]. This suggests that such halogenated compounds can circumvent typical single-resistance pathways in bacteria, which rely on efflux pumps or enzymatic degradation, and that the multi-targeted attack may likewise help overcome multidrug resistance barriers in cancer cells.

Similar to ADCs, PARP inhibitors are a triumph of targeted therapy, exploiting a specific vulnerability in cancer cells. Although anthraquinone derivatives and PARP inhibitors share a broadly similar effect pathway, which includes DNA breaks, inhibiting replication, and ultimately triggering cell death, their underlying mechanisms of action differ significantly. Anthraquinone derivatives act through more direct means, such as intercalating into DNA to prevent unwinding and replication, or generating ROS that damage DNA via oxidative stress. These mechanisms lead to widespread DNA breakage, resulting in broader toxicity and a higher likelihood of harming normal cells. In contrast, PARP inhibitors interfere with the DNA single-strand break repair pathway in tumor cells. This interference indirectly leads to more lethal DNA double-strand breaks. In cancer cells with homologous recombination repair deficiencies, such as those with BRCA mutations, these breaks cannot be repaired, ultimately causing cell death. Due to this high selectivity, normal cells with intact homologous recombination pathways can efficiently repair the DNA damage induced by PARP inhibitors, while tumor cells with the defects are selectively targeted. As a result, PARP inhibitors offer a more favorable therapeutic index compared to broad-spectrum cytotoxic agents. Currently, the PARP inhibitors approved by the U.S. FDA for the treatment of ovarian or breast cancer include olaparib, rucaparib, niraparib, and talazoparib. According to clinical data, hematologic toxicity is among the most common adverse events. A report of 29 phase II and III randomized controlled trials involving five PARP inhibitors showed that anemia was the most frequent hematologic toxicity observed [112]. Other common toxicities included neutropenia, thrombocytopenia, and gastrointestinal effects, but these side effects were considered manageable. Unlike anthracyclines, PARP inhibitors are less frequently associated with cardiovascular toxicity. Furthermore, when combined with other anticancer agents such as paclitaxel or bevacizumab, the risk of cardiotoxicity appears to be significantly reduced [113]. Resistance mechanisms differ markedly between anthraquinone-based agents and PARP inhibitors. While multidrug resistance to anthraquinones is often driven by nonspecific processes, PARP inhibitors face more diverse and well-characterized resistance routes that are closely linked to their DNA repair-targeted mechanism of action. The most prevalent is restoration of homologous recombination function, often through *BRCA1/2* reversion mutations or loss of regulators such as tumor protein 53-binding protein 1, which re-establishes DNA repair capacity [114]. Furthermore, stabilization of replication forks can protect cancer cells from fork collapse, thereby preventing cytotoxicity even in the presence of PARP inhibition [115]. Taken together, while anthraquinones resistance tends to arise from broad multidrug pathways, PARP inhibitors encounter more mechanism-specific resistance reflecting their reliance on synthetic lethality, which has important implications for combination strategies and patient stratification.

Compared with traditional chemotherapy using anthraquinone derivatives, the integration of these compounds into ADCs represents a significant advancement in precision oncology. Through targeted delivery, ADCs not only enhance the antitumor efficacy of drugs but also minimize systemic toxicity, offering a promising direction for the treatment of aggressive breast cancer subtypes such as TNBC. Notably, anthraquinone derivatives has been considered a popular payload candidate for ADCs due to its potent cytotoxicity. Tiancimycin A (TNM A), an anthraquinone-fused enediyne compound, was encapsulated in liposomes modified with trastuzumab. Through HER2-targeted immunoliposomes (ILs), the drug was more effectively concentrated at tumor sites. Compared to non-targeted liposomes or free TNM A, HER2-TNM A-ILs significantly enhanced cellular uptake and *in vivo* tumor accumulation. A single dose of 0.02 mg/kg was sufficient to inhibit the growth of HER2+ KPL-4 tumors in mice, without inducing obvious toxicity [116]. A study on the cancer treatment of ADCs of novel anthracycline derivatives mentioned that, the linker such as maleimidocaproyl-L-valine-L-citrulline-p-aminobenzoylcarbamate and maleimidocaproyl-L-phenylalanine-L-lysine-p-aminobenzoylcarbamate were covalently attached to the anthracycline scaffold via the nitrogen atom of an oxazolidine ring, typically through an enzyme-cleavable amide or carbamate bond. This design allowed for intracellular cleavage of the linker, ensuring drug release specifically within target cells. Moreover, the second oxazolidine moiety could be hydrolyzed to a 1,2-amino alcohol group, providing a means to fine-tune solubility and pharmacokinetics without compromising the drug's activation potential [117]. The structural compatibility between suitable linkers and the anthracycline scaffold provides valuable insights for further design. If two potential reactive sites exist on the anthraquinone molecule, such as carbonyl groups or functional groups on the acridone ring, one end could be employed for linker attachment while the other retains a hydrophilic functional group, potentially enhancing the compound's solubility. Meanwhile, the electronic effects of halogen substituents may modulate the pKa and chemical reactivity of anthraquinone derivatives, thereby optimizing the cleavage rate of the linker by enzyme in cells and enabling a more controlled drug release profile [118].

### 5.3 Mitochondrial-targeted therapies of anthraquinone derivatives and ROS balance

Mitochondria in cells oxidize glucose, fats, and amino acids to provide energy for cellular activities. They also function like cellular controllers, playing essential roles in processes including cell differentiation, signal transduction, and apoptosis. By inducing mitochondrial dysfunction, researchers found that breast cancer cells could undergo reprogramming and initiate metastasis in the absence of functional mitochondria. The Cancer Genome Atlas of TNBC patients further revealed differences in oxygen consumption between TNBC and other subtypes, along with reduced mitochondrial genome (mtDNA) copy number and imbalanced mtDNA sequences. These findings suggest impaired mitochondrial function in TNBC, leading to disruption of the cellular respiratory chain [119]. Previous studies have shown that tumor cells can exchange mitochondria with infiltrating lymphocytes. Tumor cells exploit this exchange to offload their defective mitochondria into lymphocytes, thereby impairing their immune function [120]. A recent study published on TNBC therapeutic strategy further revealed that, for the first time, TNBC cell lines such as 4T1 could also steal mitochondria from nearby nerve cells [121]. These mitochondria derived from neurons enhanced the function of tumor cells with mitochondrial damage. Such tumor cells typically had low oxygen consumption and poor viability, but after five days of co-culture with neurons, their metabolism and proliferation significantly recovered, likely due to the acquisition of functional mitochondria from nerve cells [121]. Therefore, disrupting the connection between cancer cells and mitochondria is critical for suppressing cancer cell metabolism and metastatic potential. A classical anthraquinone derivative, emodin, has been reported to induce mitochondrial-mediated apoptosis in gastrointestinal cancer cells by upregulating pro-apoptotic proteins B-cell lymphoma 2 (BCL2)-associated X protein and BCL2-antagonist/killer 1, changing mitochondrial membrane potential and promoting cytochrome *c* release. This cascade subsequently activates caspase-9 and caspase-3, leading to PARP cleavage and ultimately resulting in cell apoptosis [122]. Candidate derivative 1-nitro-2-acyl anthraquinone-leucine also decreased the mitochondrial membrane potential of HCT116 cancer cells in a dose-dependent manner, which is a hallmark of early cell apoptosis [123]. In this context, anthraquinone derivatives have attracted attention for their ability to induce mitochondrial-mediated apoptosis and disrupt cancer cell energy metabolism.

Since mitochondrial defects often lead to excessive ROS production, oxidative stress-induced DNA damage also significantly contributes to the cytotoxicity mechanism of anthraquinone derivatives. In normal cell function, a delicate balance is maintained between oxidation and reduction reactions, otherwise the production of ROS can break this balance by leading to oxidative damage of cellular macromolecules such as lipids, proteins, and DNA [124]. Anthraquinones have been regarded as a class of highly redox active molecules, since they can generate ROS through the redox cycle, leading to oxidative stress, DNA strand breaks and apoptosis in cancer cells [125]. Relevant studies have proven that the addition of halogens could enhance this effect. Some halogenated quinones with redox activity could cause epigenetic modifications by promoting DNA demethylation, then altering cell function and gene expression. The DNA modification under oxidative stress is often accompanied by upregulation in demethylation levels, which is closely related to the oxidative damage induced by anthraquinones in cancer cells [126]. On the other hand, cancer cells tend to show higher basal ROS levels due to metabolic abnormalities and mitochondrial dysfunction. Additional ROS released during treatment may counteract therapeutic benefits by exacerbating redox imbalance, and the ability to effectively trigger ROS release and exert pharmacological effects is also highly dose-dependent in various models, highlighting that dose control is particularly critical for achieving favorable outcomes [127]. For example, in HeLa cell models, treatment with emodin at 10, 30, and 50 μM for 15 min resulted in a dose-dependent increase in ROS levels. However, experimental data showed that 10 μM emodin exerted minimal effects on cell viability, whereas significant inhibition was observed only at 50 μM [128]. Similarly, aloe-emodin has been reported to induce ROS production in a dose-dependent manner within the range of 10–40 μM in the HepaRG cell line. The most pronounced effects were observed after 48 h of treatment at 40 μM, characterized by mitochondrial membrane protein loss and caspase cascade activation, ultimately leading to apoptosis [129]. Another anthraquinone metabolite, rhein, at 50 μM promoted the generation of ROS and calcium ions in A-549 lung cancer cells after 12–72 h of exposure, causing mitochondrial membrane potential loss [130]. Although classic anthraquinones exhibit clear pro-apoptotic activity at higher concentrations, 50 μM emodin markedly induced ROS accumulation and endoplasmic reticulum stress in animal models, thereby promoting hepatotoxicity, triggering apoptosis in L02 hepatocytes, and increasing mice mortality rate [131]. Moreover, elevated ROS measured after anthracycline exposure correlated with cardiomyocyte necrosis and decreased left ventricular ejection fraction in animal and patient samples. In biochemical assays using isolated tumor organelles, ROS formation showed apparent Km values around 103–125 μM, with dose-dependent increases in superoxide, H_2_O_2_, and hydroxyl radical signals [132]. By contrast, assays in human induced pluripotent stem cell-derived cardiomyocytes showed that even clinically relevant low-dose exposure to 1 μM of DOX triggered elevated intracellular ROS, lipid peroxidation, and apoptosis within 24 h [133]. Similarly, in isolated adult mouse cardiomyocytes, exposure to 3 μM for 15 min was sufficient to disrupt mitochondrial activity and calcium homeostasis, with early mitochondrial impairment noted at this low micromolar range [134]. As DOX is an anthraquinone-based chemotherapeutic, these observations underscore the critical importance of dose calibration in designing mitochondrial-targeted anthraquinone derivatives, as excessive ROS generation can undermine therapeutic benefits and exacerbate host toxicity.

## 6 Safety and toxicity of anthraquinone derivatives

Although anthraquinone compounds have shown superior anti-cancer activity, safety concerns and accompanying side effects caused by the drugs are still the focus of attention. Marketed anthraquinone drugs have various adverse effects due to wide application in the fields of laxatives, anti-inflammatory and anticancer. Long-term and high-dose use of anthraquinone compounds has been found to cause damage to the liver and kidney. For instance, a daily dose of anthraquinone reaching 5.44 mg/kg induced renal tubule injury in Sprague Dawley rats, while a dose exceeding 174.08 mg/kg resulted in mild hepatocyte hypertrophy and hypothyroidism in female rats [135]. A combined *in vitro* cytotoxicity and *in silico* reverse dosimetry approach demonstrated that rhein had potential hepatotoxicity according to EC_50_ value of 10 μM in HepG2/C3A and HuH-7 cell lines [136]. Exceptionally, xanthopurpurin (1,3-dihydroxyanthracene-9,10-dione) and lucidin-ω-methyl ether exhibited low toxicity on normal kidney epithelial cells along with IC_50_ of 67.89 μM and 79.01 μM [137]. In addition, halogenated anthraquinones such as 1-(4-chlorophenylthio)anthraquinone and 3-bromo-1-hydroxy-9,10-anthraquinone not only enhanced stability but also exhibited strong cytotoxicity against breast cancer cells. Meanwhile, the cytotoxicity assay results demonstrated that such compounds showed no toxic effects on normal endothelial cells, suggesting a promising anticancer therapeutic with minimized side effects [57].

As laxatives, anthraquinone compounds exert powerful effects but are accompanied by adverse reactions. Long-term abuse of anthraquinone laxatives can lead to metabolic alkalosis, low blood pressure, weight loss, and even melanosis coli [138]. Besides, diacerein (4,5-diacetyloxy-9,10-dioxoanthracene-2-carboxylic acid), an oral anthraquinone derivative used to treat osteoarthritis, has been associated with dose-dependent diarrhea. As a result, the European Medicines Agency changed the recommended dose to 50 mg per day [138,139]. Despite reports showing no genotoxicity or chromosomal aberrations associated with anthraquinone treatment, oral administration of anthraquinone during pregnancy resulted in decreased body weight and food intake in pregnant rats, and fetal viscera malformed when the dose reached 217.6 mg/kg, whereas the highest dose with no adverse effects was 21.76 mg/kg [140]. Overall, most anthraquinones are safe at the recommended dose, but potential adverse effects need to be monitored, especially in cases of chronic disease or long-term megadose treatment.

## 7 Discussion of challenge and future perspective

Breast cancer remains one of the leading causes of death among women, not only due to the disease burden itself but also because of the severe side effects of treatments such as chemotherapy. Conventional chemotherapy and targeted therapy drugs such as DOX often have serious side effects including cardiotoxicity and liver impairment. Some hormone-targeted therapies, such as tamoxifen and the HER2 inhibitor trastuzumab, develop resistance to long-term treatment. With the advancement of medical research, the pathogenic mechanisms underlying the cardiotoxicity of anthracyclines have been found to involve mitochondrial dysfunction, ferroptosis, cardiomyocyte senescence, and gut microbiota dysregulation. These emerging insights have driven the development of novel strategies, such as nano-targeted delivery systems, stem cell therapy, and multi-target regulation by natural compounds.

Although the application of anthraquinone derivatives has brought encouraging clinical outcomes to cancer patients, such as novantrone and pixuvri, a large number of halogenated anthraquinones with strong *in vitro* biological activity remain stagnant in the laboratory. Numerous challenges lie on the path from laboratory research to clinical application of these drugs and novel therapeutic approaches. From a pharmacokinetic perspective, many lead compounds fail due to poor absorption, high clearance, or rapid excretion, resulting in systemic or tissue exposure that cannot reach efficacious levels *in vivo* [141]. This has repeatedly been identified as a direct reason why promising *in vitro* active compounds lack measurable *in vivo* effects. For instance, the identification of emodin metabolites in rats using ultrahigh-performance liquid chromatography/quadrupole-orbitrap mass spectrometry revealed that its absolute bioavailability was only 3.2%. Approximately 56% of emodin was not absorbed and was primarily excreted in its prototype form through feces. The absorbed fraction was rapidly metabolized into hydroxylated and glucuronidated metabolites, thereby reducing plasma levels of free drug [142]. If certain compounds enter the body in the form of anthraquinone glycosides, they must undergo hydrolysis by intestinal bacteria and hepatic enzymes to remove the sugar moiety, thereby converting them into more lipophilic aglycones that are active and absorbable. This complex biotransformation not only alters drug exposure but may also generate a variety of secondary metabolites, leading to unpredictable pharmacokinetic behavior. Moreover, since 2000, original pharmacokinetic data on the absorption, clearance, and excretion of halogenated anthraquinones *in vivo* have been nearly absent, which stands in stark contrast to their strong *in vitro* activity. Although halogenation may improve the physicochemical properties or binding affinity of anthraquinone derivatives, the entire parent nucleus family generally suffers from pharmacokinetic limitations. If a halogenated anthraquinone exhibits spectacular *in vitro* cytotoxicity, the subsequent research focus should perhaps shift to aspects such as solubility, dissolution rate, plasma protein binding, metabolite identification, tissue distribution, and genotoxicity assessment. Only with these data can halogenated anthraquinone derivatives take a further step toward clinical research.

Lipophilic anthraquinone derivatives are readily absorbed by highly perfused organs and tend to accumulate in the liver, spleen, intestine, and adipose tissue, thereby increasing the risk of off-target exposure beyond the tumor site. To address these challenges, formulation strategies have been explored as a means to optimize delivery and pharmacokinetic performance. For cases of intestinal irritation, poor solubility, or high first-pass loss, colon-specific carriers such as chitosan conjugates and Pickering emulsions, or nanoparticle encapsulation (PEG-PLGA), may be considered to improve local or systemic exposure and reduce gastrointestinal toxicity. Rhein covalently linked with chitosan enables Pickering emulsions to achieve colon-specific release and enhance bioavailability. Moreover, compared with the free drug, PEG-PLGA nanoparticles co-loaded with emodin improve liver tumor exposure and therapeutic efficacy [143]. Beyond these approaches, bioconjugate strategies such as ADCs further extend the concept of optimized delivery by combining selective targeting with cytotoxic payloads. The advances in ADCs therapeutics have not only highlighted the value of cytotoxicity of anthraquinone derivatives but also emphasized the importance of precisely modulating oncogenic signaling pathways. In particular, the VEGF/Notch signaling axis, a crucial driver of tumor angiogenesis and progression, offers a promising target for anthraquinone-based intervention. VEGFR2, which serves as a target antigen in several ADCs like ramucirumab, suggests that anthraquinone-based small molecules may also indirectly suppress VEGF expression by modulating upstream regulators such as HIF-1α. Alternatively, they may exert multi-target regulatory effects on signaling networks by inhibiting key kinases within interconnected pathways such as PI3K/AKT and MAPK [144]. Anthraquinone derivatives, known for their potent cytotoxicity, are ideal payload candidates. Coupling these compounds with antibodies against proteins in the VEGF/Notch axis may inhibit tumor growth and metastasis, as well as disrupt tumor vasculature and microenvironmental signaling, thereby enhancing therapeutic efficacy in aggressive breast cancer subtypes.

However, the lack of in-depth research at the molecular level hinders the development of anthraquinone derivatives as anticancer agents. For instance, in both *in vivo* and *in vitro* breast cancer models, anthraquinone compounds have shown minimal involvement in the Notch signaling pathway and MCP-1, which are key signaling pathways and cytokines associated with breast cancer metastasis, especially in the highest aggressive subtype TNBC. On the other hand, breast cancer cells are more susceptible to anthraquinones-induced oxidative stress resulting in apoptosis due to their high dependence on redox balance. ROS secretion can interfere with cancer signaling pathways, blocking the cell cycle and inhibiting proliferation, which is a desired cytotoxic mechanism *in vitro* but also underlies genotoxicity and organ damage when uncontrolled *in vivo*. More than that, ROS generated by drugs at subcytotoxic doses activates transcription factors such as Snail, which in turn induce EMT, thus promoting the migration and invasion ability of cancer cells. In a study investigating DOX intervention in different breast cancer cell lines, treatment of MCF7 cells with 0.1 μg/mL for 24 h activated protein tyrosine kinase 2 phosphorylation via mtROS production, thereby enhancing cell migration and invasion. Similarly, 4T1 cells exhibited strong responses at DOX concentrations of 0.01 and 0.1 μg/mL, with markedly increased migration and EMT marker expression. Encouragingly, DOX in breast cancer mouse models was not sufficient to trigger a complete metastatic cascade [145]. Comparable investigations are still lacking for halogenated anthraquinones, highlighting the need for systematic pharmacological and toxicological studies to generate supporting data. The paradoxical dual effects of ROS may require precise dose control of anthraquinone compounds, as well as consideration of combination therapy strategies to avoid triggering the tumor transfer mechanism.

Lastly, given the ongoing challenges mentioned above, the SAR of anthraquinone derivatives should be further exploited in the research and development of new drugs. Halogenation techniques have been primarily applied in fields such as materials science, agrochemical development, and industrial chemistry for a long time. Still, the recent U.S. FDA approvals of halogen-containing small molecule drugs have brought halogen substitution back into the spotlight. Lessons learned from these drugs include positional halogen placement and halogen bond design to improve exposure and mitigate toxicity. In certain compound series, para-halogenation of aromatics, such as with F or Cl, can statistically reduce human liver microsomal clearance, whereas ortho or meta substitutions may sometimes increase clearance [146]. Thus, the substitution position is critical for optimizing metabolic stability. In drug design or molecular docking studies, if the spatial geometry of the target protein is already known, deliberately placing heavier halogens such as Cl, Br, or I at positions that can form halogen bonds with the target may enhance binding affinity and selectivity. The successful application of halogens and the resurgence of halogen-containing small molecule drugs deserve greater attention in the field of natural compound research, particularly in the context of developing novel strategies for cancer therapy. Incorporating halogenated elements into the planar anthracene structure represents a promising and inevitable strategy for optimizing the anticancer activity and selectivity of anthraquinone compounds, paving the way for a new generation of derivatives with improved therapeutic potential in breast cancer treatment, while also highlighting the importance of promoting the clinical translation of existing halogenated anthraquinone compounds with high potential.

## CRediT authorship contribution statement

**Chenyu Zhou:** Writing – review & editing, Writing – original draft, Investigation, Data curation. **Murni Nazira Sarian:** Writing – review & editing, Investigation. **Xiaohui Tong:** Writing – review & editing, Validation. **Rongchun Han:** Writing – review & editing, Data curation. **Theebaa Anasamy:** Writing – review & editing, Validation, Funding acquisition, Conceptualization, Supervision. **Hamizah Shahirah Hamezah:** Writing – review & editing, Writing – original draft, Validation, Supervision, Funding acquisition, Conceptualization.

## Declaration of competing interest

The authors declare that they have no known competing financial interests or personal relationships that could have appeared to influence the work reported in this paper.
